# Feeding preference of *Altica deserticola* for leaves of *Glycyrrhiza glabra* and *G. uralensis* and its mechanism

**DOI:** 10.1038/s41598-020-58537-y

**Published:** 2020-01-30

**Authors:** Honglei Chang, Pengyou Chen, Miao Ma

**Affiliations:** Ministry of Education Key Laboratory of Xinjiang Phytomedicine Resource Utilization, College of Life Sciences, Shihezi University, Xinjiang, 832003 The People’s Republic of China

**Keywords:** Plant sciences, Plant ecology

## Abstract

*Altica deserticola* (Coleoptera: Chrysomelidae) is a monophagous insect that feeds on, and is thus a harmful pest of, liquorice. Both adults and larvae feed on leaves, causing serious damage to leaf blades. It will even lead to the extinction of liquorice, resulting in significant economic losses. Leaf-disc tests were used to determine the feeding preference of *A. deserticola* on leaves of *Glycyrrhiza uralensis* and *G. glabra* and explore the underlying mechanism of liquorice feeding resistance to *A. deserticola* by comparing leaf hardness and thickness, cuticle thickness, and nitrogen and tannin content in the two plants. The results showed that larvae and adults have the same feeding preferences, i.e., both preferably fed on *G. uralensis*, indicating a higher resistance in this species. The hardness, thickness, and the thickness of the stratum corneum of the leaves of *G. glabra* were significantly greater than those of *G. uralensis*. Nitrogen content was higher in *G. uralensis*, while total tannin, tannic acid, and catechin content were higher in *G. glabra*. The thick cuticle and hard texture of *G. glabra* leaves may be an important physical trait for effectively resisting *A. deserticola* feeding, while high tannin and low nitrogen content may also be important.

## Introduction

*Glycyrrhiza uralensis* Fisch. ex DC. and *G. glabra* Linn. are perennial herbs of the family Leguminosae^1^. They are medicinal liquorices listed in the Chinese Pharmacopoeia^2^. Their roots and rhizomes have many functions in Chinese traditional medicine, such as relieving coughs^3^, reducing phlegm^4^, antiasthmatic^5^, protecting the liver^6^, anti-HIV^7^, and inhibiting the proliferation of cancerous cells^8^. Glabridin from the belowground organs of *G. glabra* has a skin-whitening effect^9^. It is thus favoured by medical and cosmetic industries. However, overexploitation has increasingly decreased wild resources of liquorice, and both abovementioned species are endangered in China^10,11^. The contradiction between supply and demand of these plants has been increasingly prominent. In recent years, cultivated liquorice has effectively alleviated this contradiction. However, during cultivation, frequent outbreaks of pests considerably decrease the yield and quality of liquorice^12^.

*Altica deserticola* Latreille (Coleoptera: Chrysomelidae) is a monophagous insect and the most harmful pest of liquorice, feeding on its leaves^13^. It usually breaks dormancy in April, enters dormancy at the end of September, and produces 3 or 4 generations per year^14^. Both the adults and larvae feed on liquorice leaves, causing serious damage to the leaf blades, and thereby weakening the photosynthetic capacity of the plants and reducing the yield and quality of liquorice roots and rhizomes^15^. Only chemical control via spraying chemical pesticides is presently adopted by farmers to combat this beetle, which easily leads to the presence of pesticide residues in the medicinal materials of the plants. Therefore, searching for liquorice varieties with higher resistance to *A. deserticola* will boost cultivation enthusiasm and industrial development of liquorice. According to our field observation, we found that there was much higher population density of the beetle and more severe damage caused by the pest in *G. uralensis* than *G. glabra* fields. Whether the difference in pest density and damage was caused by variation in the biological characteristics of the two liquorices or by differences in local climate or cultivation management measures, such as different water or fertilizer management strategies, among different plants remains unclear.

Some physical and chemical characteristics of leaves can usually affect the feeding behaviour or intensity of herbivorous insects^16^. In the ordinary course of events, the insects tended to feed on tender, soft, nitrogen-rich leaves, and avoided those with poor palatability or phytotoxins^17^. In the present paper, the feeding preference of *A. deserticola* for *G. uralensis* and *G. glabra* leaves was investigated and the hardness, thickness, cuticle thickness, and nitrogen and tannin content of the two liquorices were compared to reveal the underlying mechanism in the differences in feeding intensity between the two species. Our findings may provide theoretical reference for breeding liquorice varieties with increased resistance to the beetle.

## Materials and Methods

### Investigation in liquorice field

On 10th July 2018, we selected two adjacent 1-ha plots in *G. uralensis* and *G. glabra* fields with the same soil conditions and agricultural regime in Shawan Farm (45°12′N, 85°28′E). A five-point sampling method was used for randomly selecting five quadrats (10 m × 10 m) along the diagonals and at the centre point of each field. Number of liquorice individuals and damaged plants (with at least one hole or notch on its leaves), density of adult and larval beetles, and damage rate of the liquorice individuals in each quadrat were counted, and the average values from five sampling plots were calculated.

### Plant and insect samples

*A. deserticola* adults were collected from a population of *Glycyrrhiza aspera* Pall in the eastern suburb of Shihezi, Xinjiang, China (44°32′N, 86°10′E). All adults were housed in a light incubator under 12 h of illumination at 25 °C and 12 h of darkness at 20 °C (light intensity, 200 μ mol•m^−2^•s^−1^) and were fed with fresh leaves of *G. aspera* daily. The fertilized eggs were collected from leaves of *G. aspera*, incubated in a light incubator, and hatched in ~6 days. The larvae were also fed fresh leaves of *G. aspera* daily; they pupated in ~15 days and emerged into adults after 6–8 days. To avoid the effect of leaf age and cultivation condition, including soil, climate, water, and fertilizer factors on the physical and chemical characteristic of the leaves and feeding preference of the beetle, the fully expanded fresh leaves of *G. uralensis* and *G. glabra* at the same age were collected from the position of the fifth leaf from the top of the two liquorices cultivated at the Liquorice Resource Center of Shihezi University, Shihezi, Xinjiang, China (44°18′N, 86°05′E), and these two liquorice species were cultivated under the same conditions. The mean annual precipitation and temperature in the region were 125–207.7 cm and 6.5–7.2 °C, respectively.

### Feeding preference of *A. deserticola* for the two liquorices

**Figure 1:**
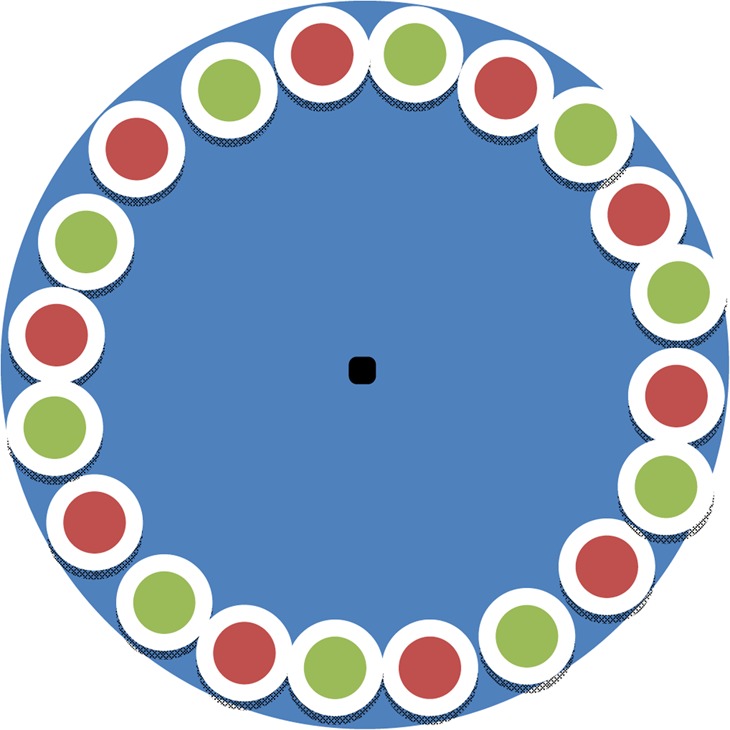
Arrange pattern of leaf discs and pest in a petri dish. Note: the green and red circles represent leaf discs of *Glycyrrhiza uralensis* and *G. glabra*, respectively; the black dot represents the larval or adult *Altica deserticola*.

A leaf-disc method was used to determine the feeding preference of the adults or larvae of *A. deserticola* for the leaves of *G. uralensis* and *G. glabra*. The leaves of 30 different plants for each species were randomly selected. They were rinsed with clean water, dried with filter paper, and 1-cm-diameter discs were obtained with a disc cutter punch. Ten leaf discs of each species (total of 20 leaf discs) were placed annularly and alternately in a petri dish (9 cm diameter, Taixing Mingtai Scientific Instruments and Equipment Co., Ltd., Jiangsu, China) over a wet sponge covered with filter paper (Ø9 cm, Hangzhou Special Paper Co., Ltd., Hangzhou, China). Thirty healthy second-instar larvae (hatched for ~6 days) or adults with the same body size were selected and placed at the centre of the filter paper surrounded with the leaf discs, one per dish after starvation for 5 h, and a total of 30 petri dishes were used for the method shown in Fig. 1. Petri dishes with leaf discs but without *A. deserticola* were used as controls. The larvae or adult beetles were allowed to feed in each experiment for ~24 h. The leaf discs were then pressed and dried, and the leaf area consumed (%) was determined using a HP Scanjet 5300C scanner (Hewlett-Packard, Loveland, CO, USA) and Adobe Photoshop CS6 (Adobe, San Jose, CA, USA). The leaf area consumed was considered to be the percentage of the total damaged area to the total area of the 10 discs.

### Mechanical and chemical properties of leaves of the two liquorices

#### Leaf hardness

Penetrability of the leaves of *G. uralensis* and *G. glabra* (maximum penetrability value represented the leaf hardness) were detected using a texture analyser (TA. XT plus, Stable Micro Systems, Godalming, Surrey, UK) with its accompanying software Exponent 32. Measurements were taken under the following settings: HDP/CH detection base, SMS P/2 N sharp probe, 2 mm•s^−1^ speed before puncture, 1 mm•s^−1^ speed during puncture, 10 mm•s^−1^ speed after puncture, and 20 g puncture trigger value. Thirty healthy and fully expanded leaves of each liquorice were randomly selected, and each leaf was tested three times to obtain average values.

#### Leaf thickness and cuticle thickness

Healthy, fully expanded leaves of *G. uralensis* and *G. glabra* from 10 individual plants of each liquorice were cut into small pieces (1 cm × 0.5 cm) and placed in FAA solution (70% alcohol: glacial acetic acid: formaldehyde = 18:1:1) for 48 h. Transverse sections of the leaves (8 μm thick) were prepared using conventional paraffin sectioning^18^. The sections were stained with safranin and fast green, sealed with optical resin, observed under a light microscope (Olympus BX51, Olympus Optical, Tokyo, Japan), and photographed with an Olympus DP70 system. Leaf and cuticle thickness of the adaxial and abaxial surface were measured by Motic Images Advanced 3.2 (Motic, Hong Kong), calculated their average value.

#### Leaf nitrogen content

We randomly selected 150 plants and collected one healthy and fully expanded leaf from each plant for 30 leaves per sample. The leaf samples were dried to constant weight, pulverized with a grinder (HAY-201, Hao You Electrical Appliance Factory, Zhongshan, China), and sieved through a 1.98-mm mesh, and then a 0.1-g sample was accurately weighed. Nitrogen content of the leaves was measured using a Kjeldahl apparatus (K9840; Haineng Instrument Co., Ltd., Jinan, China) after digestion with sulfuric acid–hydrogen peroxide (H_2_SO_4_–H_2_O_2_) as described by Kirk^19^. Five samples were tested five times and their average values were calculated.

#### Tannin content

Leaf samples of the two species were dried to constant weight, pulverized with a grinder (HAY-201), and sieved through a 1.98-mm mesh and 0.2 g of leaf powder was accurately weighed. The total tannin content was determined using the Folin–Ciocalteu procedure^20^ and tannic acid was used as a standard. The content of tannic acid^21^, ellagic acid^22^, gallic acid^23^, and catechin^24^ were detected by high-performance liquid chromatography (Agilent 1200; Agilent Technologies, CA, USA). Five samples of each plant were tested, and their average value was calculated. Setting conditions were as follows:

Tannic acid: the mobile phase contained solvent A: 0.07% acetic acid 15% and solvent B: methanol 85%, isocratic elution. The flow rate was 0.5 mL•min^−1^ and the volume injected was 10 µL. The temperature of the column was 25 °C, and UV detector was set at a wavelength of 275 nm.

Ellagic acid: the mobile phase contained solvent A: 0.1% acetic acid and solvent B: acetonitrile. The gradient was 12–20% B for 16 min, 20–25% B for 4 min. The flow rate was 1.0 mL•min^−1^ and the volume injected was 20 µL. The temperature of the column was 30 °C, and UV detector was set at a wavelength of 265 nm.

Gallic acid: the mobile phase contained solvent A: 0.1% acetic acid and solvent B: acetonitrile. The gradient was 5–7.5% B for 10 min. The flow rate was 1.0 mL•min^−1^ and the volume injected was 10 µL. The temperature of the column was 25 °C, and UV detector was set at a wavelength of 267 nm.

Catechin: the mobile phase contained solvent A: 0.1% acetic acid 68% and solvent B: methanol 32%, isocratic elution. The flow rate was 1 mL•min^−1^ and the volume injected was 10 µL. The temperature of the column was 30 °C, and UV detector was set at a wavelength of 254 nm.

## Data Analysis

The SPSS 19.0 software (IBM Corp., New York, USA) was used to analyse the data. Differences in leaf area consumed (%), leaf hardness and thickness, cuticle thickness, leaf nitrogen and tannin content between the two liquorices were analysed using a T-test. Multiple comparison analysis was used for comparing the differences in content of the four kinds of tannins for each liquorice species. The charts were produced using Origin 2016 (OriginLab, Hampton, USA).

## Results

### Population density of *Altica deserticola* and the damage rate of liquorices

The average density of adult and larval populations in the *G. uralensis* field reached 13.8 and 3.2/m^2^, respectively (Table 1). Those in the *G. glabra* fields were only 1.8 and 0.124/m^2^, respectively (Table 1). The average damage rates of *G. uralensis* and *G. glabra* were 86.7% and 2.36%, respectively (Table 1).

**Table 1:** Population density of *Altica deserticola* and the damage rate of liquorice species.

Liquorice species	Quadrats	No. of Plants	Adult beetle density (No. per m^2^)	Larva beetle density (No. per m^2^)	The damage rate of liquorice (%)
*Glycyrrhiza uralensis*	U1	1823	16	5	90
	U2	1865	15	4	86
	U3	1852	15	2	86.2
	U4	1831	10	3	84
	U5	1812	13	2	87.3
*G. glabra*	G1	1863	1	0	3
	G2	1825	2	0.12	2.1
	G3	1822	2	0.3	3.3
	G4	1834	1	0.2	1.4
	G5	1826	3	0	2

Note: “U” stands for the *G. uralensis* quadrat, and “G” stands for the *G. glabra* quadrat.

### Comparison on the consumed amount of leaves by *A. deserticola* between the two liquorices

**Figure 2:**
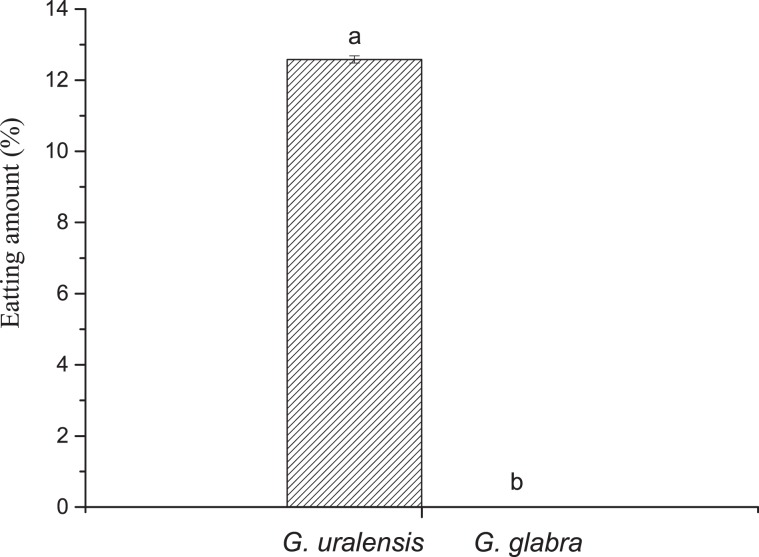
The percentages (%) of the leaf area of *Glycyrrhiza glabra* and *G. uralensis* consumed by adults of *Altica deserticola*. Different letters denote significant differences between the two plants. The error bar represents the standard deviation (*P* < 0.01).

**Figure 3:**
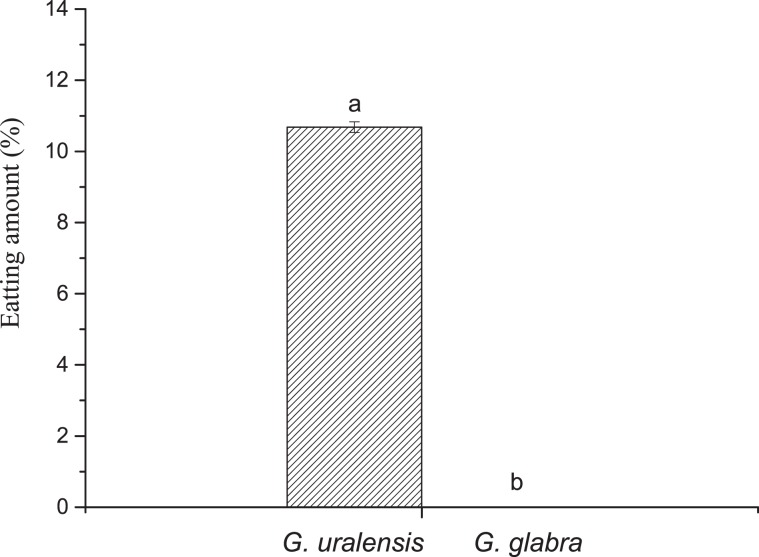
The percentages (%) of the leaf area of *Glycyrrhiza glabra* and *G. uralensis* consumed by larvae of *Altica deserticola*. Different letters denote significant differences between the two plants. The error bar represents the standard deviation (*P* < 0.01).

Both adults and larvae of *A. deserticola* only fed on the leaves of *G. uralensis*, while all the leaf discs of *G. glabra* in culture dishes remained intact. The consumption percentage of adults to leaf area reached 12.58% (Fig. 2), and the consumption percentage of larvae to leaf area reached 10.68% (Fig. 3).

### Comparison of leaf hardness

**Figure 4:**
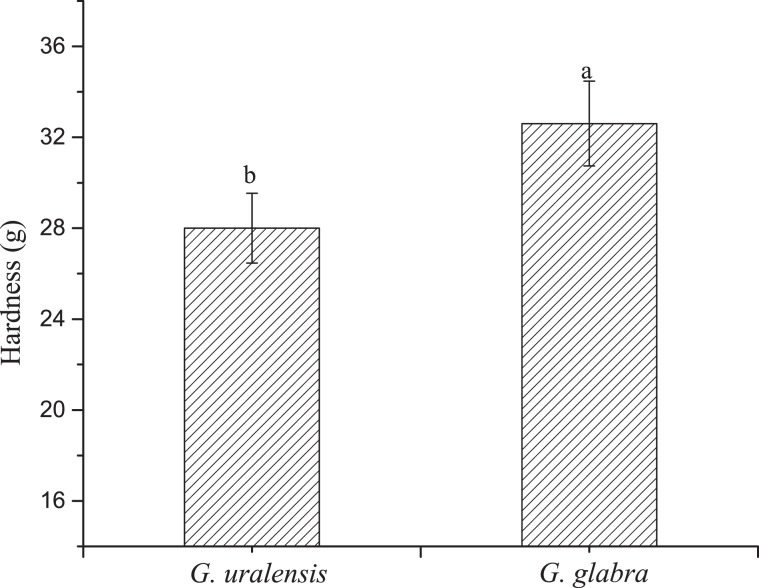
Leaf hardness of two liquorice (*Glycyrrhiza*) species. Means with different letters are significantly different. The error bar represents the standard deviation (F = 1.109, df = 58, *P* = 0.003).

The leaves of *G. glabra* are leathery with a hard texture, while those of *G. uralensis* are soft textured (Fig. 4). There was a significant difference in the hardness value of the leaf between the two plants.

### Comparison of blade and cuticle thickness

The leaves of *G. glabra* were significantly thicker than those of *G. uralensis* (Table 2; *P* = 0.01). The leaf cuticle thickness on the adaxial and abaxial side in *G. glabra* was also significantly greater than that in *G. uralensis* (Table 2; *P* = 0.002).

**Table 2:** Comparison of leaf thickness and cuticle thickness between two species of liquorice.

Indexes (μm)		F	df	Glycyrrhiza uralensis	*G. glabra*
Leaf thickness		0.94	8	228.07 ± 3.58b	250.49 ± 3.5a
Leaf cuticle thickness	adaxial	0.89	8	2.23 ± 0.083b	3.02 ± 0.073a
	abaxial	0.64	8	1.73 ± 0.083b	2.65 ± 0.144a

Different letters in the same row indicate significant difference (*P* < 0.05). Data are presented as means ± standard deviation.

### Comparison of nitrogen content

**Figure 5:**
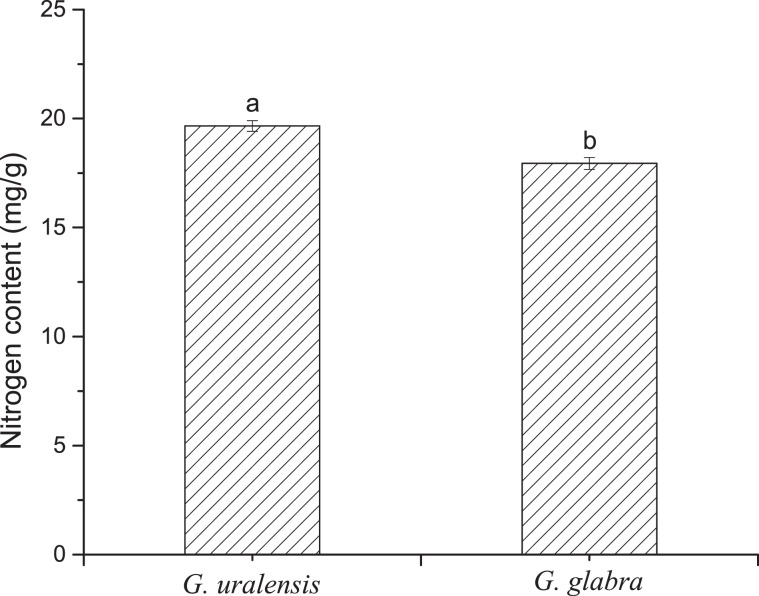
Leaf nitrogen content of two liquorice (*Glycyrrhiza*) species. Means with different letters are significantly different. The error bar represents the standard deviation (F = 0.146, df = 8, *P* = 0.002).

The leaf nitrogen content in *G. uralensis* was higher than that in *G. glabra* (Fig. 5), and there was a significant difference in the nitrogen content between the two liquorices (*P* = 0.002).

### Comparison of tannin contents

**Figure 6:**
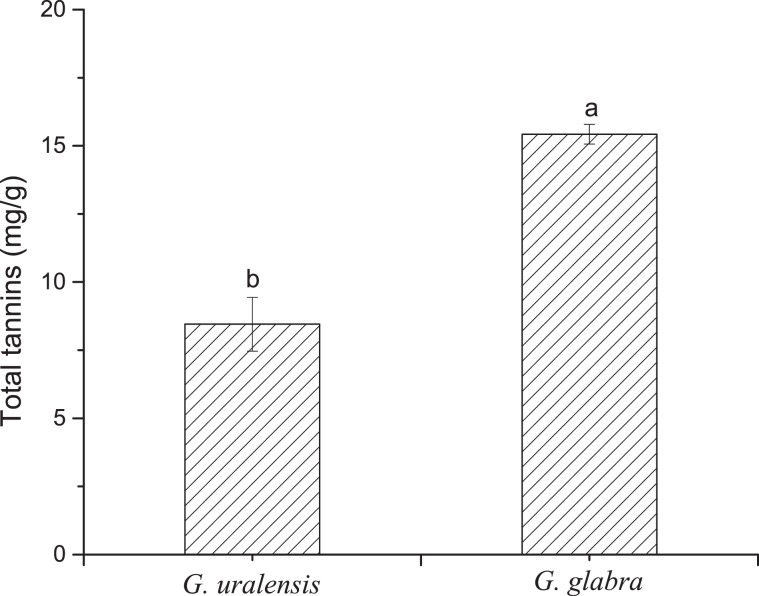
Total tannin content in the leaves of two liquorice (*Glycyrrhiza*) species. Means with different letters are significantly different. The error bar represents the standard error (F = 6.348, df = 8, *P* = 0.003).

**Figure 7:**
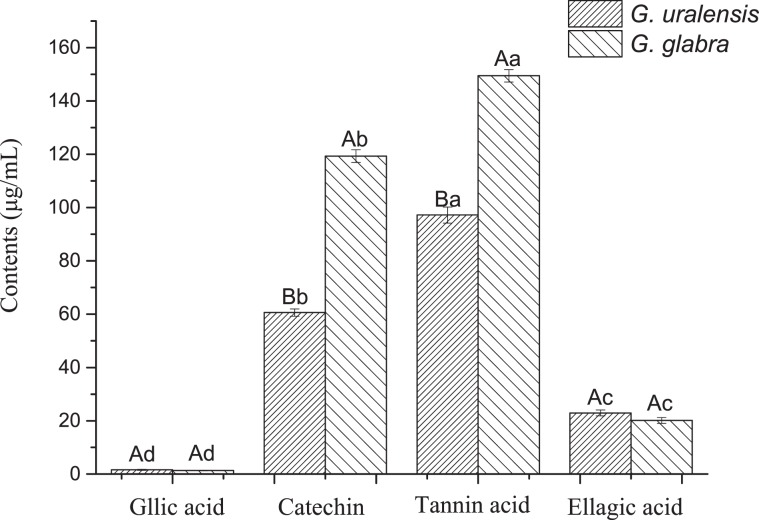
The content of four kinds of tannins in the leaves of two liquorice (*Glycyrrhiza*) species. Different capital letters denote significant differences between means of the columns (P < 0.01), and different lowercase letters denote significant differences between means of the four kinds of tannins in the same liquorice species (P < 0.01).

The total tannin content in the leaves of *G. glabra* was significantly higher than that of *G. uralensis* (Fig. 6; *P* = 0.003). In both species, the tannic acid content was the highest followed by catechin with both accounting for >92% of the total tannin content in *G. glabra* and 86% of that in *G. uralensis*. Hence, we concluded that they were the main constituents of tannins in liquorice leaves. The content of gallic acid and ellagic acid in the leaves of the two liquorices was relatively low, especially in *G. glabra* leaves (7% of the total tannin content; Fig. 7).

## Discussion

Consumption of plant leaves by insects causes loss of photosynthetic organs, reduces net photosynthetic rate and biomass accumulation, and thus inhibits plant growth^25,26^. *A. deserticola* is a pest, whose various generations overlap and insects with different developmental stages coexist. It will cause extreme damage to *G. uralensis* leaves, since both the adults and the larvae are dependent on these leaves for food. When devastating outbreaks of the pest occur, there are often 10 ~ 20 adults and larvae of the insects found on a liquorice plant, which eat the leaves reducing the plant’s photosynthetic ability causing the liquorice to wither and die, resulting in a significant decrease in the production of roots and rhizome. E.g. *G. uralensis* planted in Shawan Farm was investigated on 10th July 2018, and ~85% of its leaves had been eaten by *A. deserticola* (Table 1). Therefore, it is of great scientific and economic significance to study the feeding preferences and its mechanisms of *A. deserticola* on liquorice leaves.

In the present study, we found that *A. deserticola* preferred eating *G. uralensis* leaves over those of *G. glabra* when the two types of plants coexisted under the same conditions, which eliminated the influence of environmental differences on the feeding preferences of *A. deserticola*. This was consistent with the results of our previous field observation. Therefore, the feeding preference of *A. deserticola* for leaves of *G. glabra* and *G. uralensis* is likely to be related to the physical and chemical properties of the leaves themselves.

Physical properties of the leaves, including hardness, thickness, and the presence of trichomes and wax on the surface can significantly affect feeding behaviour of the insects^27^. Huang^28^ showed that tea varieties with thick leaves were of better resistance to *Myllocerinus aurolineatus* Voss than those with thin leaves. Hoffman and Rao^29^ reported that the hardness degree of the host plant leaves significantly affected the behaviours of *Oulema melanopus*, which preferred softer leaves. In the present study, some physical characteristics of the leaves of the two liquorices tested were selected for analysis. Combined with the feeding preference of *A. deserticola*, it can be seen that *A. deserticola* preferred to eat thin and soft leaves with thin cuticles.

Nitrogen was recognized as the most important limiting nutrient for herbivorous insects. The C:N ratios of the herbivores were considerably lower than those of their potential foods, but the insect required nutrient-rich resources to rapidly build nutrient-rich bodies^30^. To meet such high nitrogen demand, the insect must feed on nitrogen-rich plants. In the present study, we found the nitrogen content in the leaves of *G. uralensis* was significantly higher than that in *G. glabra*, which was consistent with the feeding preference of *A. deserticola*. This indicated that nitrogen content in leaves was an important factor affecting the feeding behaviour of *A. deserticola*, i.e. the higher the nitrogen content in leaves the higher the feeding preference of the pest.

Tannins are secondary metabolites of plants. They are natural polyphenolic compounds and widely exist in leguminous plants. Previous studies reported that leaves of liquorice plants contained tannins, and tannic acid, catechin, ellagic acid, and gallic acid were four major components^31–34^. We determined the content of total tannins and their four components in the leaves of the two liquorices. Our results showed that the content of total tannins in the leaves of *G. glabra* was significantly higher than that in those of *G. uralensis*. Tannin is an important defensive substance in plants against their pests, which lengthens insect developmental times^35^. Sun^36^ found a significant negative correlation between the tannin content of leaves in different poplar varieties and the feeding intensity of *Saperda populnea* (Coleoptera: Cerambycidae). Therefore, tannins affect the palatability of insects and thus the feeding preferences of phytophagous insects^37^. *G. glabra* leaves have high tannin content, which resulted in poor palatability. This may be another reason why *A. deserticola* only feeds on the leaves of *G. uralensis*.

In summary, the feeding preference of *A. deserticola* for the leaves of the two liquorices was the result of a combination of various factors. The physical and chemical characteristics, such as leaf hardness, leaf thickness, cuticle thickness, and nitrogen and tannin content of leaves, may be important factors affecting the feeding preference of *A. deserticola*. Tannic acid was the tannin component with the highest content in the leaves of *G. uralensis* followed by catechin. The content of these two substances in the leaves of *G. glabra* was significantly higher than those in the leaves of *G. uralensis*. Therefore, we speculate that the differences in content of the two tannin components may be one of the reasons for the feeding preference of the beetles.

To gain more accurate results, we should add these two substances to the leaves of *G. uralensis* and investigate whether there is a difference in leaf consumption between the added group and the non-added groups under the same conditions. Colour and volatile compounds of plant leaves could obviously affect the feeding behaviours of some other insects^38,39^. Thus, whether the differences in colour and volatile compounds of the two plant leaves significantly affect the feeding behaviour of the beetle should be further studied.
